# High rate of complete responses to immune checkpoint inhibitors in patients with relapsed or refractory Hodgkin lymphoma previously exposed to epigenetic therapy

**DOI:** 10.1186/s13045-016-0363-1

**Published:** 2016-11-30

**Authors:** Lorenzo Falchi, Ahmed Sawas, Changchun Deng, Jennifer E. Amengual, Donald S. Colbourn, Emily A. Lichtenstein, Karen A. Khan, Lawrence H. Schwartz, Owen A. O’Connor

**Affiliations:** 1 Center for Lymphoid Malignancies, Division of Hematology/Oncology, Department of Medicine, Columbia University Medical Center, 51 West 51st Street, Suite 200, New York, NY 10019 USA; 2 Department of Radiology, Columbia University Medical Center, 180 Fort Washington Avenue, New York, NY 10032 USA

**Keywords:** Hodgkin lymphoma, Pembrolizumab, Nivolumab, Azacitidine, Epigenetic therapy

## Abstract

Options for patients with relapsed or refractory (R/R) classical Hodgkin lymphoma (cHL) after brentuximab vedotin (Bv) and autologous stem cell transplantation (ASCT) are limited. Immune checkpoint inhibitors (ICI) are active in this population but rarely induce complete response (CR). Ten patients with R/R cHL after ASCT and Bv received pembrolizumab (*n* = 8) or nivolumab (*n* = 2). Five had been previously exposed to 5-azacitidine on a phase 1 study. Among nine evaluable patients, seven (78%) achieved CR, one partial response, and one reduction of tumor burden. All five patients who had received 5-azacitidine prior to ICI achieved CR, while only two of four who did not receive prior 5-azacitidine achieved CR. At a median follow-up of 9.9 months [0.5–14.3], eight patients are alive and five are still receiving treatment. We documented an unprecedented CR rate after ICI in patients with R/R cHL. We hypothesize that hypomethylating agents might have an immune priming effect and enhance the efficacy of ICI.

**Electronic supplementary material:**

The online version of this article (doi:10.1186/s13045-016-0363-1) contains supplementary material, which is available to authorized users.

## To the editor

Patients with classical Hodgkin lymphoma (cHL) refractory to, or relapsed after, autologous stem cell transplantation (ASCT) and brentuximab vedotin (Bv) have limited treatment options and represent an unmet medical need [1].

The immune checkpoint receptor programmed death (PD)-1 is expressed on T cells and causes reversible anergy when engaged by its ligands after antigen recognition by the T cell receptor [2]. Expression of PD-ligand (L)1 or PD-L2 is utilized by tumors to escape immune surveillance [3]. cHL exhibits a genetically determined overexpression of PD-L1/PD-L2 due to recurrent amplification of 9q24.1 [4], and thus may be exquisitely sensitive to PD-1 blockade. The anti-PD-1 monoclonal antibodies nivolumab and pembrolizumab, two immune checkpoint inhibitors (ICI), are highly active in patients with relapsed or refractory (R/R) cHL but induced complete response (CR) only in a minority of them [5, 6]. The hypomethylating agent (HMA) 5-azacitidine was shown to induce expression of retroviral genes in tumor cells and trigger a T cell-mediated response, thus potentially synergizing with ICI [7]. We report our experience with ICI in ten patients with R/R cHL, five of whom were previously exposed to 5-azacitidine.

Eight patients received pembrolizumab (2 mg/kg every 3 weeks) and two nivolumab (3 mg/kg every 2 weeks). Response was evaluated with fluorodeoxyglucose-positron emission tomography/computerized tomography (PET/CT) according to the 2014 Lugano criteria [8]. Patients with new or persistent lesions were allowed to continue on therapy if they had disease and/or symptom control, without unacceptable toxicity.

**Fig. 1:**
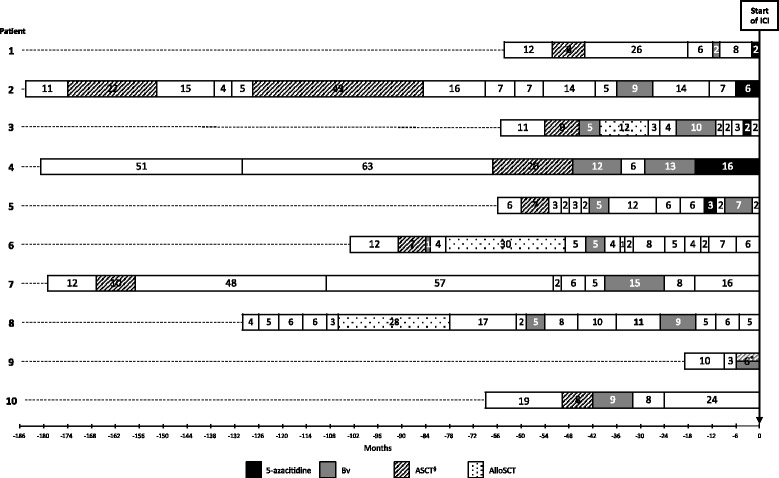
Previous therapies and respective duration of disease control. For each patient, *bar length* and *number* represent the time to next treatment (in months). *Section sign* includes salvage chemotherapy followed by high-dose chemotherapy and ASCT; *Asterisk* indicates Bv induction followed by ASCT consolidation and Bv maintenance. Abbreviations: *ICI* immune checkpoint inhibitor, *Bv* brentuximab vedotin, *ASCT* autologous stem cell transplantation, *AlloSCT* allogeneic stem cell transplantation

The median number of prior treatments was 11 [3–16] and 80% of patients had received ≥7 lines of therapy. All patients had received ASCT and Bv (Additional file 1: Table S1). Five patients had been previously treated with 5-azacitidine in combination with romidepsin within a phase 1 clinical trial (NCT01998035) (Additional file 2: Table S2). Median duration of 5-azacitidine therapy was 3 months [2–16]. Three patients received it immediately prior to ICI, the other two within 14 months of initiating ICI (Fig. 1).

**Fig. 2:**
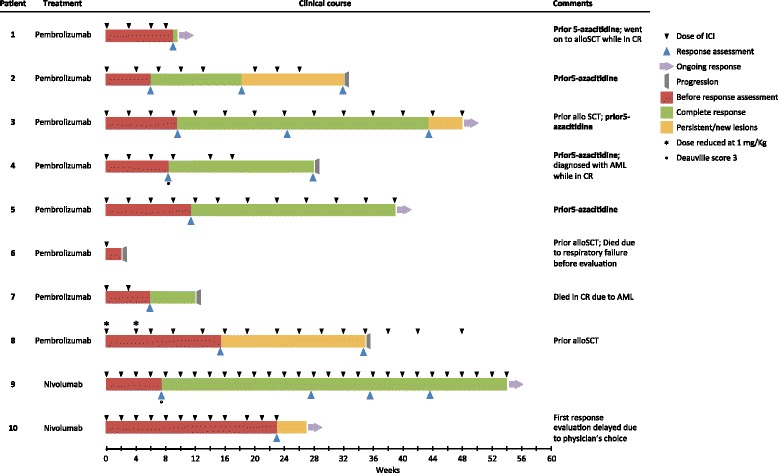
Type of immune checkpoint inhibitor, treatment duration, dose intensity, and type and duration of response. For each patient, the *bar length* indicates the amount of time spent in a specific (color-coded) disease state. Abbreviations: *AlloSCT* allogeneic stem cell transplantation, *MDS* myelodysplastic syndrome, *AML* acute myeloid leukemia, *CR* complete response, *ICI* immune checkpoint inhibitor

The median treatment duration was 25 weeks [2–54]. Two treatment delays lasting >1 week were due to lack of insurance coverage and diagnosis of myelodysplastic syndrome (MDS) (Fig. 2). There were six grade ≥3 adverse events (AE): one patient had an infusion reaction, one had thrombocytopenia and was later diagnosed with MDS, and another developed chronic myelomonocytic leukemia (CMML) right after treatment initiation. The latter two patients had been exposed to radiation and alkylating agents and developed acute myeloid leukemia (AML), fatal in one case. One patient with chronic kidney disease developed a non-steroidal anti-inflammatory drug-related interstitial nephritis, in resolution at the time of pembrolizumab initiation. One patient developed hypotension, hypoxia and bilateral pulmonary infiltrates after the first infusion and died 2 weeks later of multi-organ failure, despite broad-spectrum antibiotics and high-dose steroids. This patient had pre-existent depressed left ventricular ejection fraction (20%), recurring pericardial effusion, post-radiation fibrosis and bronchiectasis, and recent pneumonia. Grade 1–2 AE are summarized in Additional file 3: Table S3.

Nine patients were evaluable for response. Seven (78%) achieved CR and one partial response. One patient had a significant reduction of all tumor sites and developed a new liver lesion. All five patients who had been exposed to 5-azacitidine containing therapy achieved a CR, whereas only two of the four who did not receive prior 5-azacitidine achieved CR (Fig. 2). After a median follow-up of 9.9 months [0.5–14.3] eight patients were alive and five were still receiving treatment after 23, 39, 48, 48, and 54 weeks. The patient with a new liver lesion had stable disease, no new symptoms, and no significant toxicity, thus continued to receive ICI therapy. Three patients discontinued therapy: one transitioned to alloSCT while in CR after 9 weeks of therapy and remains in remission 1 year later; one discontinued while in CR after 17 weeks, due to transformation of CMML into AML; and the third patient discontinued treatment due to confirmed progression and lack of clinical benefit after 32 weeks (Fig. 2).

To the best of our knowledge, this is the first report to suggest clinical synergy between epigenetic therapy and ICI in patients with cHL. In this very heavily pre-treated cohort, ICI showed impressive clinical efficacy with a CR rate (CRR) of nearly 80%. In phase 1 trials in patients with R/R cHL, nivolumab (*n* = 23) induced an overall response rate (ORR) and CRR of 87% and 17%, respectively [5], and pembrolizumab (*n* = 31), at a dose of 10 mg/kg, an ORR of 65% and CRR of 16% [6]. With the limitations of comparing separate, small series, our cohort appears to include more extensively pre-treated patients (Additional file 1: Table S1 and [5, 6]). Yet, most of them achieved CR early after treatment.

All five patients previously exposed to 5-azacitidine obtained PET/CT-negative CR. Recent studies suggested that HMA can induce up-regulation of endogenous retroviral genes in tumor cells. Endocellular sensors of viral double-strand RNA then trigger an interferon-β-mediated T cell response. Moreover, in a pre-clinical melanoma model, 5-azacitidine was found to sensitize cells to ipilimumab, another ICI [7]. This agent may, therefore, have a “priming” effect on the immune system and maximize the response to ICI. The impact of 5-azacitidine might be harder to assess in patient N. 5, as he received the drug about 1 year prior to starting ICI. Unlike 5-azacitidine, histone deacetylase inhibitors have not been shown to synergize with ICI. However, because 5-azacitidine was administered in combination with romidepsin in our patients, we cannot exclude that additional romidepsin-mediated modulation of gene expression might have further potentiated the effects of ICI, thus contributing to the high CRR. Other limitations of our retrospective study include the small sample size, the retrospective nature of the study, the heterogeneous duration and timing of HMA therapy, and the use of different ICI. Nevertheless, the uniquely high CRR cannot be explained by ICI therapy alone and may be in part due to synergy with HMA. The combination of epigenetic therapy and PD-1 blockade is being tested prospectively at our institution.

## Additional files

Additional file 1: Table S1. Characteristics of the patients included in the present report and the studies from Ansell and colleagues and Armand and colleagues. (DOCX 90 kb)
Additional file 2: Table S2. Treatment schedule of combined 5-azacitidine and romidepsin (phase 1 study, NCT01998035). (DOCX 57 kb)
Additional file 3: Table S3. Adverse events observed during immune checkpoint inhibitor therapy. (DOCX 15 kb)

## Abbreviations

AE: Adverse event
AML: Acute myeloid leukemia
ASCT: Autologous stem cell transplantation
Bv: Brentuximab vedotin
cHL: Classical Hodgkin lymphoma
CMML: Chronic myelomonocytic leukemia
CR: Complete response
CRR: Complete response rate
HMA: Hypomethylating agent
ICI: Immune checkpoint inhibitor(s)
MDS: Myelodysplastic syndrome
ORR: Overall response rate
PD-1: Programmed death-1
PD-L1, PD-L2: PD-ligand 1, PD-ligand 2
PET/CT: Fluorodeoxyglucose-positron emission tomography/computerized tomography
R/R: Relapsed or refractory
