# Supplementary material for: High rate of complete responses to immune checkpoint inhibitors in patients with relapsed or refractory Hodgkin lymphoma previously exposed to epigenetic therapy
Source: J Hematol Oncol. 2016 Nov 30;9:132. doi: 10.1186/s13045-016-0363-1 (PMC5129196; doi:10.1186/s13045-016-0363-1)
Supplement: Additional file 1: Table S1. — Characteristics of the patients included in the present report and the studies from Ansell and colleagues and Armand and colleagues. (DOCX 90 kb) [file 13045_2016_363_MOESM1_ESM.docx]

**Supplementary Table 1. Characteristics of the patients included in the present report and the studies from Ansell and colleagues and Armand and colleagues.**

| **Characteristic** | **Present report**  **(N = 10)** | **Ansell et al.**  **(N = 23)** | **Armand et al.**  **(N = 31)** |
| --- | --- | --- | --- |
| Male : Female ratio | 6:4 (60:40) | 12:11 (52:48) | 18:13 (58:42) |
| Median age, years [range] | 35 [24-57] | 35 [20-54] | 32 [20-67] |
| Race, N (%) |  |  | NR |
| White | 6 (60) | 20 (87) |  |
| African-American | 2 (20) | 2 (9) |  |
| Other | 2 (20) | 1 (4) |  |
| ECOG PS, N (%) |  |  | NR |
| 0 | 3 (30) | 6 (26) |  |
| 1 | 6 (60) | 17 (74) |  |
| 2 | 1 (10) | 0 |  |
| Histological Subtype, N (%) |  |  |  |
| Nodular sclerosis | 9 (100)* | 22 (96) | 30 (97) |
| Mixed cellularity | 0 (0) | 1 (4) | 1 (3) |
| Extranodal lymphoma, N (%) | 8 (80) | 4 (17) |  |
| ≥4 lines of prior therapy, N (%) | 9 (90) | 15 (65) | 21 (68) |
| Prior ASCT, N (%) | 10 (100) | 18 (78) | 22 (71) |
| Prior Bv, N (%) | 10 (100) | 18 (78) | 31(100) |
| Prior alloSCT, N (%) | 3 (30) | NA | NR |
| *one case was not evaluable due to crash artifacts present in the tissue biopsy. Abbreviations: ECOG PS, Eastern cooperative study group performance status; ASCT, autologous stem cell transplant; Bv, brentuximab vedotin; alloSCT, allogeneic stem cell transplant; NA, not applicable (alloSCT was an exclusion criterion in the study); NR, not reported. | | | |
