# Supplementary material for: High rate of complete responses to immune checkpoint inhibitors in patients with relapsed or refractory Hodgkin lymphoma previously exposed to epigenetic therapy
Source: J Hematol Oncol. 2016 Nov 30;9:132. doi: 10.1186/s13045-016-0363-1 (PMC5129196; doi:10.1186/s13045-016-0363-1)
Supplement: Additional file 2: Table S2. — Treatment schedule of combined 5-azacitidine and romidepsin (phase 1 study, NCT01998035). (DOCX 57 kb) [file 13045_2016_363_MOESM2_ESM.docx]

**Supplementary Table 2. Treatment schedule of combined 5-azacitidine and romidepsin (phase 1 study, NCT01998035)**

| **Patient N.** | **Oral 5-azacitidine** | **Intravenous romidepsin** | **Cycle duration** |
| --- | --- | --- | --- |
| **1** | 300 mg daily, days 1-14 | 10 mg/ms, days 8 and 15 | 28 days |
| **2** | 200 mg daily, days 1-14 | 10 mg/ms, days 8 and 15 | 28 days |
| **3** | 300 mg daily, days 1-14 | 10 mg/ms, days 8 and 15 | 28 days |
| **4** | 200 mg daily, days 1-14 | 10 mg/ms, days 8 and 15 | 28 days |
| **5** | 100 mg daily, days 1-14 | 10 mg/ms, days 8 and 15 | 28 days |
| **11** | 300 mg daily, days 1-21 | 10 mg/ms, days 8, 15, and 22 | 35 days |

Note: since these patients were enrolled in a phase 1, dose-finding study, they belonged to different dose cohorts, featuring different dosing schedules.
