# Supplementary material for: High rate of complete responses to immune checkpoint inhibitors in patients with relapsed or refractory Hodgkin lymphoma previously exposed to epigenetic therapy
Source: J Hematol Oncol. 2016 Nov 30;9:132. doi: 10.1186/s13045-016-0363-1 (PMC5129196; doi:10.1186/s13045-016-0363-1)
Supplement: Additional file 3: Table S3. — Adverse events observed during immune checkpoint inhibitor therapy. (DOCX 15 kb) [file 13045_2016_363_MOESM3_ESM.docx]

**Supplementary Table 3. Adverse events observed during immune checkpoint inhibitor therapy**

|  | **Averse events** | |
| --- | --- | --- |
|  | **Grade 1-2** | **Grade 3-5** |
| **Patient 1** | Infusion reaction |  |
| **Patient 2** |  | Infusion reaction |
| **Patient 3** | Infusion reaction |  |
| **Patient 4** | Infusion reaction, hypothyroidism | Thrombocytopenia, MDS° |
| **Patient 5** |  |  |
| **Patient 6** |  | Respiratory failure |
| **Patient 7** | Hypothyroidism* | CMML*° |
| **Patient 8** |  | AKI*§, infusion reaction |
| **Patient 9** | Infusion reaction, diarrhea |  |
| **Patient 10** | Hypothyroidism |  |
| *pre-existing ICI therapy, °evolved into acute myeloid leukemia; §resulting in stable chronic kidney disease. Abbreviations: ICI, immune checkpoint inhibitor; MDS, mylodysplastic syndrome, CMML, chronic myelomonocytic leukemia; AKI, acute kidney injury. | | |
